# In Vitro and In Vivo Bone-Forming Effect of a Low-Molecular-Weight Collagen Peptide

**DOI:** 10.4014/jmb.2307.07017

**Published:** 2023-11-20

**Authors:** Jae Min Hwang, Mun-Hoe Lee, Yuri Kwon, Hee-Chul Chung, Do-Un Kim, Jin-Hee Lee

**Affiliations:** Health Food Research and Development, NEWTREE Co., Ltd., Seoul 05604, Republic of Korea

**Keywords:** Low-molecular-weight collagen peptide, collagen hydrolysate, Gly-Pro-Hyp, bone remodeling, MC3T3-L1 cells, Sprague–Dawley rats

## Abstract

This study reveals that low-molecular-weight collagen peptide (LMWCP) can stimulate the differentiation and the mineralization of MC3T3-E1 cells in vitro and attenuate the bone remodeling process in ovariectomized (OVX) Sprague–Dawley rats in vivo. Moreover, the assessed LMWCP increased the activity of alkaline phosphatase (ALP), synthesis of collagen, and mineralization in MC3T3-E1 cells. Additionally, mRNA levels of bone metabolism-related factors such as the collagen type I alpha 1 chain, osteocalcin (OCN), osterix, bone sialoprotein, and the Runt family-associated transcription factor 2 were increased in cells treated with 1,000 μg/ml of LMWCP. Furthermore, we demonstrated that critical bone morphometric parameters exhibited significant differences between the LMWCP (400 mg/kg)-receiving and vehicle-treated rat groups. Moreover, the expression of type I collagen and the activity of ALP were found to be higher in both the femur and lumbar vertebrae of OVX rats treated with LMWCP. Finally, the administration of LMWCP managed to alleviate osteogenic parameters such as the ALP activity and the levels of the bone alkaline phosphatase, the OCN, and the procollagen type 1 N-terminal propeptide in OVX rats. Thus, our findings suggest that LMWCP is a promising candidate for the development of food-based prevention strategies against osteoporosis.

## Introduction

Osteoporosis is characterized by high bone fragility in which the imbalance between bone formation and resorption leads to a decrease of the bone mineral density (BMD). It is a noncurable disease that afflicts a large part of the population worldwide [1]. Therefore, the prevention of osteoporosis must be achieved before bone further deteriorates. Bone degradation can be delayed through the activation of bone generation or the obstructing of bone resorption. Several drugs can regulate the bone remodeling process, including bisphosphonates, selective estrogen receptor modulators, parathyroid hormone, and sclerostin [2]. However, these treatments have safety issues or are characterized by serious side effects (such as increased micro-fracture accumulations, infections, stroke or cardiovascular events) [3]. Thus, it is crucial to develop safer treatments that can facilitate bone formation.

Collagen is a crucial constituent of the skin, cartilage, tendons, and bones. Its function is to maintain their functionality and structure. Collagen is the main component of the extracellular matrix (ECM), which is linked to important cellular processes such as the regulation of cell adhesion and proliferation, the regulation of growth factors’ effectiveness, and differentiation [4]. The deposition of collagen can lead to ECM synthesis and bone reinforcement stimulation.

Through an enzyme-mediated reaction, collagen produces collagen hydrolysate or collagen peptides [5]. Collagen hydrolysate can exert various beneficial effects, including antiaging, antiobesity, and wound healing effects [6]. Clinical data have also revealed a positive effect of collagen hydrolysate on osteoporosis or bone formation [7, 8]. In these studies, collagen hydrolysate was shown to improve the hematological factors related to bone remodeling and increase the levels of BMD in humans. Collagen tripeptide has also been shown to stimulate the healing of the fracture of the femur and tibia in vivo [9, 10]. Moreover, Liu *et al*. have investigated the differentiating effect of hydrolyzed fish collagen on bone marrow mesenchymal stem cells [11], while the intake of hydrolyzed collagen [12] and cod gelatin [13] has been shown to increase the BMD of ovariectomized rats. We believe that collagen peptides, which are known to be safe to eat and have a low possibility of causing allergic reactions [14], could potentially stimulate the preosteoblastic cells and the microarchitecture of the femur and the lumbar vertebrae in ovariectomized rats.

We have previously demonstrated wrinkle reduction as well as hydration and elasticity enhancement of the human skin because of the oral administration of a low-molecular-weight collagen peptide (LMWCP) containing Gly-Pro-Hyp at 3% w/w and collagen tripeptide at over 15% w/w [15]; these effects are mediated by the promotion of the synthesis of type I collagen and of hyaluronic acid in skin fibroblasts [16, 17]. Moreover, our study revealed that orally consumed LMWCP can alleviate osteoarthritis progression in an anterior cruciate ligament transection model in rabbits by promoting ECM synthesis [18]. Based on these experiments, it was reasonable to explore the effect of LMWCP on other tissues such as bones, which also contain collagen. Therefore, we investigated the in vitro and in vivo effects of LMWCP on bone remodeling.

## Materials and Methods

### Test Material

In this study, we used LMWCP supplied by NEWTREE, Co., Ltd. LMWCP is a collagen hydrolysate using the skin of *Pangasius hypophthalmus* and proteases. It contains Gly-Pro-Hyp (3%) and tripeptide (over 15% w/w). LMWCP was dissolved in distilled water before use and was kept in a 4°C refrigerator for storage. The study was approved by Institutional Animal Care and Use Committee (Approval No.: IV-RB-02-2012-39).

### Cell Culture and Mineralization Assay

The newborn mouse calvaria preosteoblast cells (MC3T3-E1) were purchased from the American Type Culture Collection (USA) and were cultured in alpha minimum essential medium (α-MEM; Gibco BRL Co., USA) supplemented with 10% (v/v) fetal bovine serum (FBS; Gibco) and 1% (v/v) penicillin/streptomycin (Gibco) at 37°C, in the presence of 5% CO_2_. For the induction of osteoblastic differentiation, MC3T3-E1 cells were incubated in an osteogenic induction medium (α-MEM containing 10% FBS, 10 mM β-glycerophosphate, and 50 μg/mL ascorbic acid).

Calcium mineralization in MC3T3-E1 cells was evaluated by an alizarin red S staining. MC3T3-E1 cells were fixed in paraformaldehyde and the culture was stained with 2% alizarin red S. To quantify matrix mineralization, 10% (w/v) cetylpyridinium chloride was added to the culture medium and incubated to dissolve and release calcium-bound alizarin red. The absorbance of the released alizarin red was measured at 550 nm using an Infinite M200 PRO microplate reader (Tecan Trading AG, Switzerland).

### Assessment of Alkaline Phosphatase (ALP) Activity and Collagen Deposition

The ALP activity in osteoblasts was measured using an alkaline phosphatase assay kit (ab83369; Abcam, UK). ALP activity was estimated based on the optical absorbance measured at 405 nm. Total protein content was measured with a Bradford protein assay kit (5000201, Bio-Rad, USA). P-nitrophenyl phosphate (Sigma-Aldrich, USA) was used as the substrate to evaluate the ALP activity. The absorbance was measured at 595 nm and normalized to the total protein.

Collagen deposition was quantified using sirius red (VB-3017-3; VitroVivo Biotech, USA). Cell layers were fixated with Bouin’s fluid (ScyTek Laboratories, USA), followed by a staining with the picro-sirius red solution. Subsequently, the solution was removed and the cultures were washed with 0.01 M hydrochloric acid. To perform quantitative analysis, the stained material was dissolved in 0.1 N sodium hydroxide and was measured at 550 nm.

### Real-Time-Quantitative Polymerase Chain Reaction (RT-qPCR)

Total RNA was isolated from cells using a total RNA prep kit (RP101-050, BIOFACT, Republic of Korea), and the complementary deoxyribonucleic acid (cDNA) was synthesized from 1 μg of the total RNA of each sample using the PrimeScript RT reagent kit (RR037A, TaKaRa, Japan). RT-qPCR for each gene was performed using the Power SYBR Green PCR Master Mix (4368577, ThermoScientific, USA) through the Quant Studio 1 real-time PCR system (Applied Biosystems, USA). The RT-qPCR reaction was as follows: initial denaturation at 95°C for 10 min, followed by 40 cycles of denaturation at 95°C for 15sec, and annealing at 60°C for 60sec. Real-time data were assimilated in the final extension step. All reactions were performed in triplicate, and data were represented as the fold changes relative to the control. Glyceraldehyde-3-phosphate dehydrogenase (GAPDH) served as an endogenous control. The sequences of the primers used are presented in Table 1.

### Animals and Treatments

Female Sprague–Dawley rats (8-weeks-old) were purchased from Orientbio (Republic of Korea) to investigate the in vivo effect of LMWCP on osteoporosis. Rats were allowed to acclimatize for 7 days and were maintained in a controlled condition facility (temperature: 22°C ± 2°C; humidity: 50% ± 10%; ventilation frequency: 10 times/h; light/dark cycle: 12/12 h; IACUC Approval No.: IV-RB-02-2012-39). The acclimatized rats were ovariectomized (OVX) and were used in our experiment after one week of recovery. The rats were randomly assigned into three groups (*n* = 10), while LMWCP (400 mg/kg) or vehicle (distilled water) was orally administered daily for 12 weeks. At the end of the experiment, the rats were fasted and anesthetized for the collection of their femurs, lumbar vertebrae, and blood for further analysis. No significant adverse events were observed.

### Micro-Computed Tomography (micro-CT) and Immunohistochemistry

To investigate the impact of LMWCP on bone morphometric factors, femurs and lumbar vertebrae were fixed in 4% formalin, and micro-CT (SkyScan 1173; Bruker, Belgium) was conducted (instrument S/N: 16N05068; hardware version: A; software version: 1.6; source type: Hamamatsu 130/300; camera: flat panel sensor; pixel size: 50 μm; camera XY ratio: 1; inclination lifting: 0.757 μm/mm). BMD, bone volume fraction (BV/TV), specific bone surface (BS/BV), bone surface density (BS/TV), trabecular thickness (Tb. Th), trabecular number (Tb. N), trabecular separation (Tb. Sp), structure model index (SMI), and connectivity density (Conn. D) were estimated. BV/TV and BS/BV are the ratios of the segmented bone volume to the total volume and the ratio of the segmented bone surface to the segmented bone volume, respectively. SMI is an indicator of the structure of trabeculae and Conn. D indicates the degree of connectivity of trabeculae normalized by TV [19].

For the undertaking of immunohistochemistry for collagen type I alpha 1 chain (Col1A1), the slices were prepared as previously described [20]. The primary antibody (collagen type I polyclonal antibody) was applied for 1 h at a 1:500 dilution (14695-1-ap; Proteintech, IL, USA) according to manufacturer’s protocol, and the sections were subsequently incubated with a secondary antibody using an EnVision kit (K5007; Dako, USA).

### Hematological Analysis

Blood samples were collected during the autopsy and centrifuged for 10 min (3,000 rpm) after the 30 min of the coagulation process. The retrieved sera were analyzed for the following markers using kits: procollagen type 1 N-terminal propeptide (P1NP; LS-F10555, LSBio, USA), osteocalcin (OCN; LS-F5375, LSBio, USA), C-telopeptide of collagen type 1 (CTX-1; MBS9141384, MBS, USA), tartrate-resistant acid phosphatase (TRAP; MBS3803474, MBS, USA), bone alkaline phosphatase (BAP; MBS703336, MBS, USA), and ALP (ab285274, Abcam).

### Statistical Analysis

All experimental results are expressed as the mean ± standard deviation of values obtained in at least three separate tests. Student’s *t*-test was used to analyze the statistically significance of the differences in the data. Differences with a *p*-value of < 0.05 were considered as statistically significant.

## Results

### LMWCP-Treated MC3T3-E1 Cells Exhibit Increased ALP Activity, Bone Mineralization, and Collagen Content Than the Nontreated Ones

We used the MC3T3-E1 cell line (that has similar characteristics to human osteoclasts in terms of their proliferation, differentiation, and mineralization) in order to evaluate the effect of LMWCP. First, we investigated the viability of MC3T3-E1 cells after a treatment with our experimental material, LMWCP. The cytotoxicity of LMWCP was evaluated by exposing the cells to 3, 5, 10, 30, 50, 100, 300, 500, or 1,000 μg/ml for 24 and 48 h. LMWCP provoked no significant changes on the MC3T3-E1 cell viability at the assessed concentrations (Fig. S1).

**Fig. 1:**
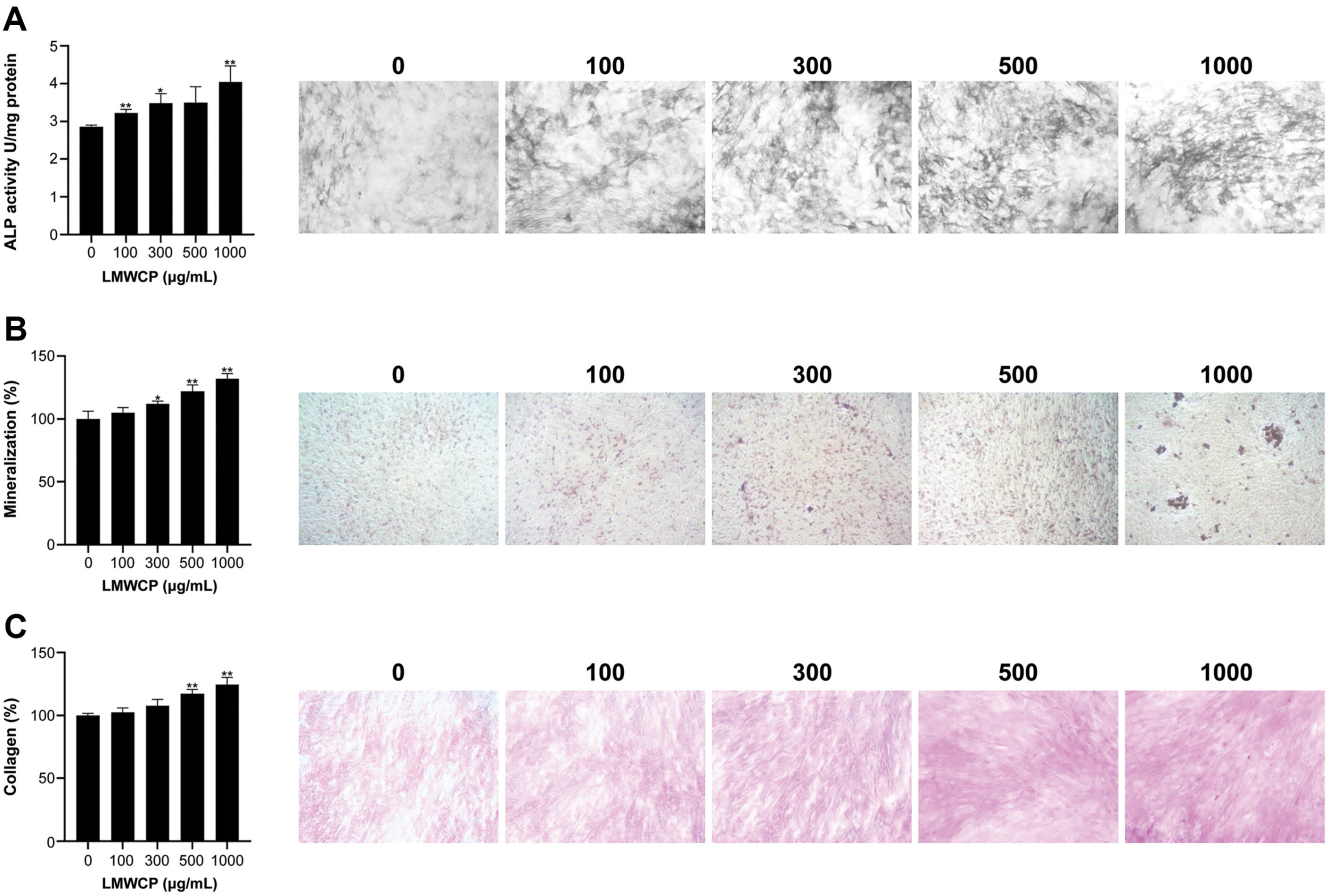
LMWCP stimulates the ALP and increases the mineralization and the collagen secretion of MC3T3-E1 cells. MC3T3-E1 cells were incubated in an osteogenic induction medium supplemented with various concentrations of LMWCP (100, 300, 500, and 1,000 μg/mL) for 14 days. (**A**) The ALP activity determination and the staining of the MC3T3-E1 cells took place after 14 days of osteogenic differentiation in the presence of LMWCP. (**B**) Analysis of the mineralization and alizarin red S staining after 14 days of osteogenic differentiation in the presence of LMWCP. (**C**) Analysis of collagen secretion and Sirius red staining after 14 days of osteogenic differentiation in the presence of LMWCP. Data represent the mean values of three independent experiments; *, *p* < 0.05; **, *p* < 0.01 (when compared with the control group).

ALP, which is located on the cellular membrane of osteoblasts, can be easily detected at the outer membrane and the mineralized tissue. Moreover, ALP acts as a marker of osteoblast activity [21] and can explain the early cell differentiation phase of osteoblastic cells [22]. As the administered concentration of LMWCP increased, the ALP was gradually activated (Fig. 1A).

The formation of a bone nodule is an important factor that can explain the differentiation of osteoblasts [23]. Therefore, we evaluated the mineralized nodule formation and its calcification using alizarin red staining (Fig. 1B). Our histological data show that the number of red-stained particles grows as the LMWCP concentration increases. The quantitative value of mineralization also indicated that higher concentration of the administered LMWCP enhanced the bone mineralization compared with the control group.

Collagen is a major component of connective tissues such as the tendons, the cartilage, and the bone. Type I collagen is the major matrix protein in the bone that is synthesized by osteoclasts [24]. To detect whether the LMWCP can stimulate the differentiation of osteoblasts or not, the collagen secretion was measured using a Sirius red staining. As Fig. 1C indicates, MC3T3-E1 cells deposited collagen when they were treated with LMWCP in a dose-dependent manner.

### LMWCP Has an Effect on the mRNA Levels of Osteogenic Factors in MC3T3-E1 Cells

**Fig. 2:**
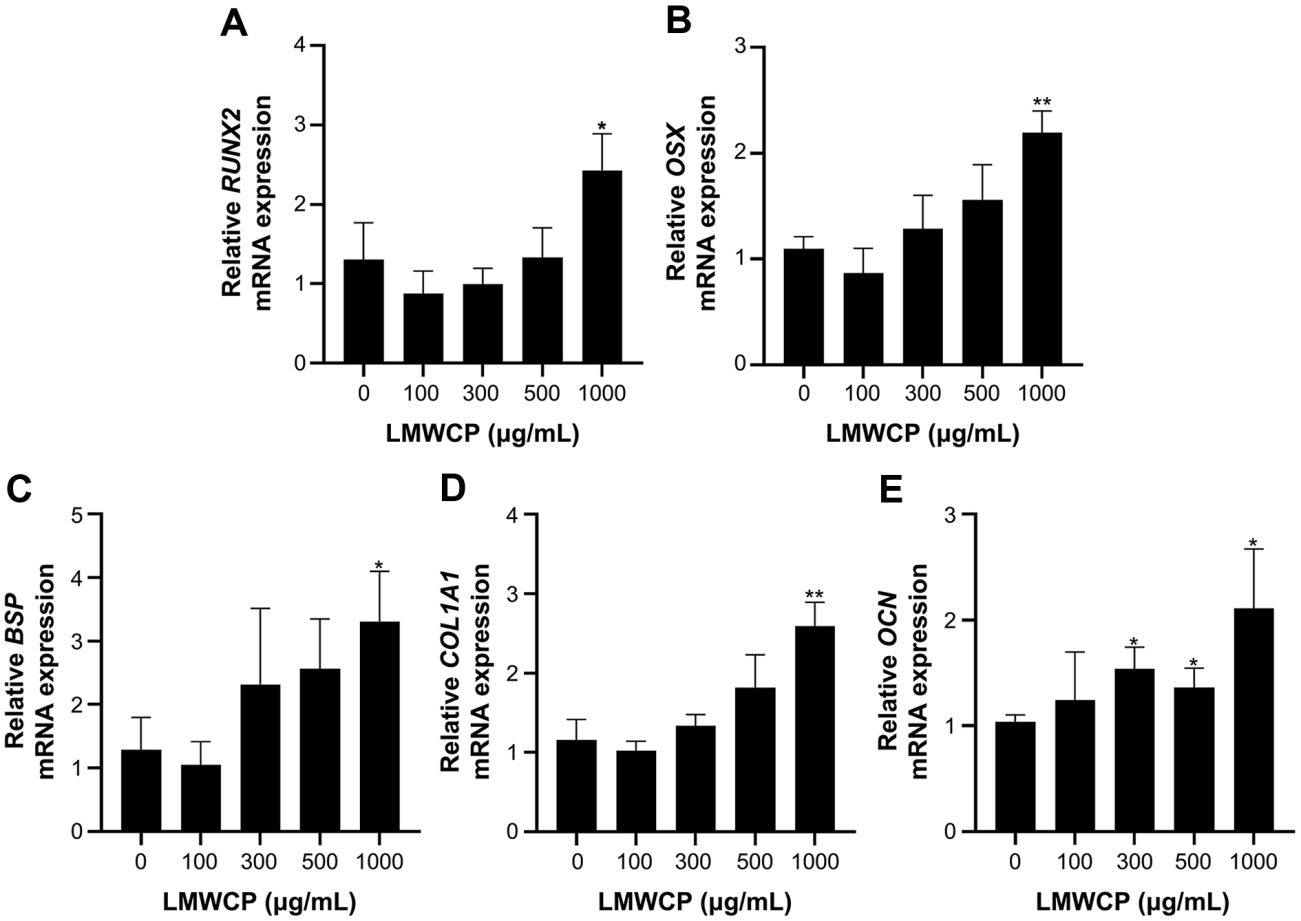
mRNA expression levels of six osteogenesis-related genes in MC3T3-E1 cells after 14 days of an incubation in an osteogenic induction medium supplemented with various concentrations of LMWCP (100, 300, 500, and 1,000 μg/mL). (**A**) Expression of RUNX2. (**B**) Expression of OSX. (**C**) Expression of BSP. (**D**) Expression of *COL1A1*. (**E**) Expression of OCN. Data represent the mean values of three independent experiments; *, *p* < 0.05; **, *p* < 0.01 (when compared with the control group).

Upregulated expression of osteogenic genes was revealed by our RT-qPCR data (Fig. 2). The cells were exposed to 100, 300, 500, 1000 μg/ml of LMWCP. The Runt family-associated transcription factor 2 (Runx2) is an early transcription factor that plays a vital role in the formation of preosteoblasts. Osterix (OSX), which is the downstream regulator of Runx2, is also a transcription factor that stimulates genes during the differentiation of preosteoblasts into mature cells [25]. Both Runx2 and OSX mRNA expressions were increased after treatment with LMWCP (Fig. 2A and 2B). In addition, bone sialoprotein (BSP), which is associated with the induction of mineralization [26], was found to be significantly upregulated in the 1,000-μg/ml LMWCP-treated group (Fig. 2C). Finally, the mRNA expression levels of bone specific matrix proteins such as Col1A1 and OCN were also found to follow similar patterns of increase (Fig. 2D and 2E). Based on these findings, one could speculate that LMWCP has osteogenic characteristics when administered to MC3T3-E1 cells, as it can increase the expression of osteoblast-related genes.

### LMWCP Improves the Levels of BMD and the Trabecular Microarchitecture of the Femur in OVX Rats

We subsequently observed the effect of LMWCP (400 mg/kg) on the trabecular microarchitecture of the femur of OVX rats. There was no significant difference in terms of body weight between the examined groups (Fig. S2). After ovariectomy, the rats belonging to the OVX and OVX + LMWCP-treated groups had more weight than those belonging to the sham group. Previous studies on OVX rats have reported similar trends [12, 27]. Thus, we can infer that the OVX model was well established.

**Fig. 3:**
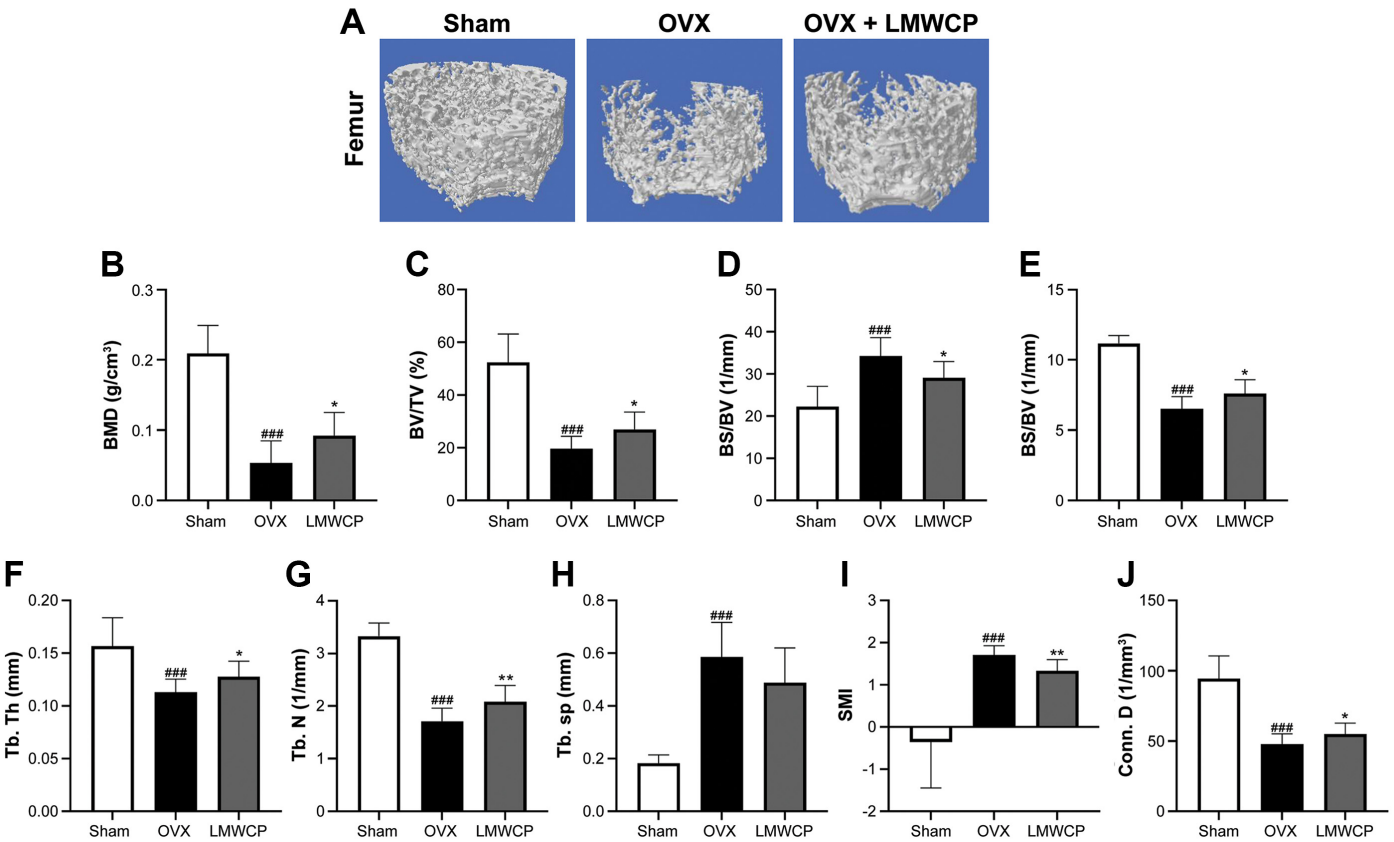
Effect of LMWCP on three-dimensional (3D) micro-CT models of the structural thickness of the right femur in OVX rats. (**A**) 3D micro-CT reconstructed images of the right femur. Quantification of the BMD (**B**), the BV/TV (**C**), the BS/BV (**D**), the BS/TV (**E**), the Tb. Th (**F**), the Tb. N (**G**), the Tb. Sp (**H**), the SMI (**I**), and the Conn. D (**J**) of sham, OVX, and OVX + LMWCP (at different LMWCP concentrations) rats. Data represent the mean values of three independent experiments; ###, *p* < 0.001 (when compared with the sham group); **, *p* < 0.01; ***, *p* < 0.001 (when compared with the OVX group).

Micro-CT was used for the elucidation of the effect of LMWCP on the trabeculae of the femurs. As Fig. 3A indicates, the OVX + LMWCP group built denser trabeculae formations compared to OVX group. The data of the histomorphometric parameters are summarized in Fig. 3B-3J. The administration of LMWCP significantly increased the BMD, BV/TV, BS/TV, Tb. Th, Tb. N, and Conn. D. Meanwhile the BS/BV was decreased after a treatment with LMWCP. Therefore, we can safely conclude that LMWCP suppresses the trabecular loss, both qualitatively and quantitatively.

### BMD Levels and the Trabecular Microarchitecture of the Lumbar Vertebrae of OVX Rats are Enhanced by LMWCP

**Fig. 4:**
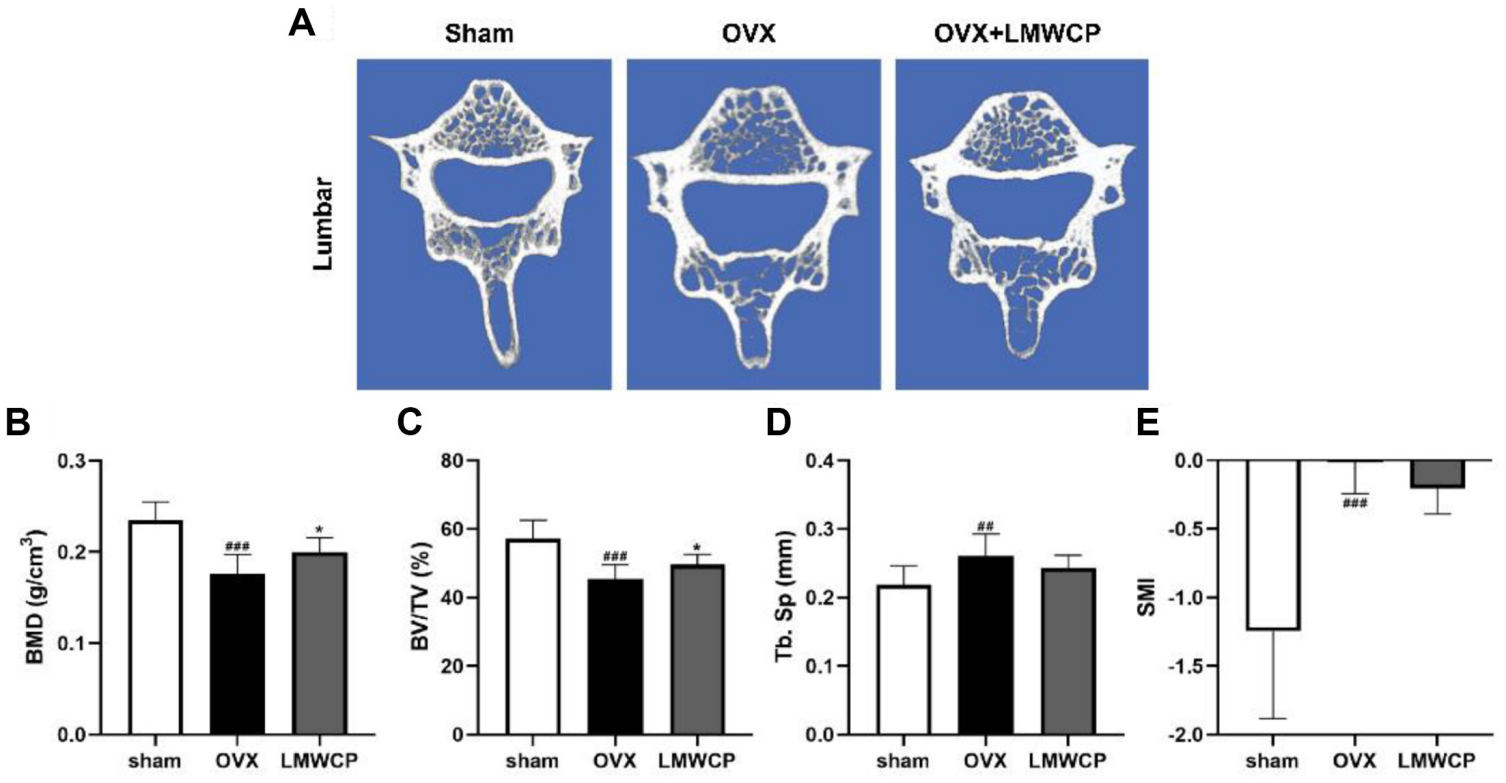
Effect of LMWCP on 3-D micro-CT models for the structural thickness of a lumbar vertebra in OVX rats. (**A**) 3D micro-CT reconstructed images of a lumbar vertebra. Quantification of the BMD (**B**), the BV/TV (**C**), the Tb. Sp (**D**), and the SMI (**E**) of sham, OVX, and OVX + LMWCP (at different LMWCP concentrations) rats. Data represent the mean values of three independent experiments; ##, *p* < 0.01; ###, *p* < 0.001 (when compared with the sham group); *, *p* < 0.05 (when compared with the OVX group).

Subsequently, the parameters of the trabecular microarchitecture were examined to validate the effect of LMWCP on the lumbar vertebrae of OVX rats. Fig. 4A presents the effect of LMWCP on the density of the lumbar vertebrae. In addition, parameters such as the BMD, BV/TV, and Tb. Sp were found altered after a treatment with LMWCP (Fig. 4B-4E). Based on these data, one can conclude that the LMWCP has an osteogenic effect on the lumbar vertebrae of OVX rats.

### Collagen Synthesis Is Upregulated in Both the Femur and the Lumbar Vertebrae of LMWCP-Treated OVX Rats

**Fig. 5:**
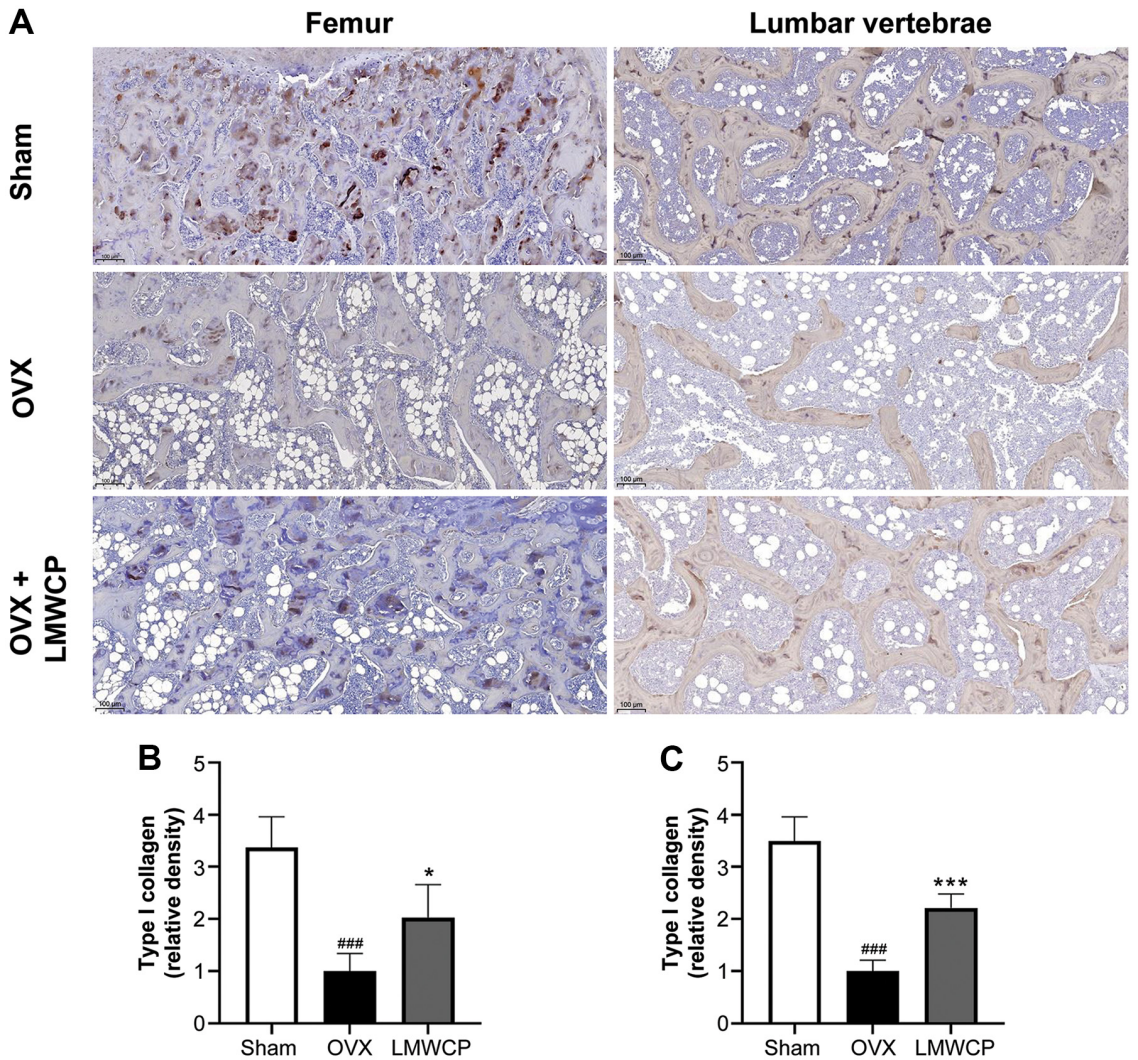
Effect of LMWCP on the immunohistochemically staining of type I collagen in the femur and the lumbar vertebrae. (**A**) Representative images of the undertaken immunohistochemical staining of the femur and the lumbar vertebrae of sham, OVX, and OVX + LMWCP rats, with the use of antibodies against the bone formation marker, type I collagen. (**B**) Quantitative results of the type I collagen formation in the sham, the OVX and the OVX + LMWCP (at different LMWCP concentrations) rats. Data represent the mean values of three independent experiments; ###, *p* < 0.001 (when compared with the sham group); *, *p* < 0.05; ***, *p* < 0.001 (when compared with the OVX group).

The deposition of collagen in the femur and the lumbar vertebrae was demonstrated through immuno-histochemistry. Collagen is the matrix component of the bone and is synthesized through ossification [28]. As shown in Fig. 5A, collagen (brown) was not synthesized when only ovariectomy took place, while formation of collagen was detected in the OVX + LMWCP group. The graph shows significant differences between the OVX and the OVX + LMWCP groups (Fig. 5B).

### Bone Metabolism Marker Levels in the Serum of OVX rats Were Found Reduced When the LMWCP Was Administered

**Fig. 6:**
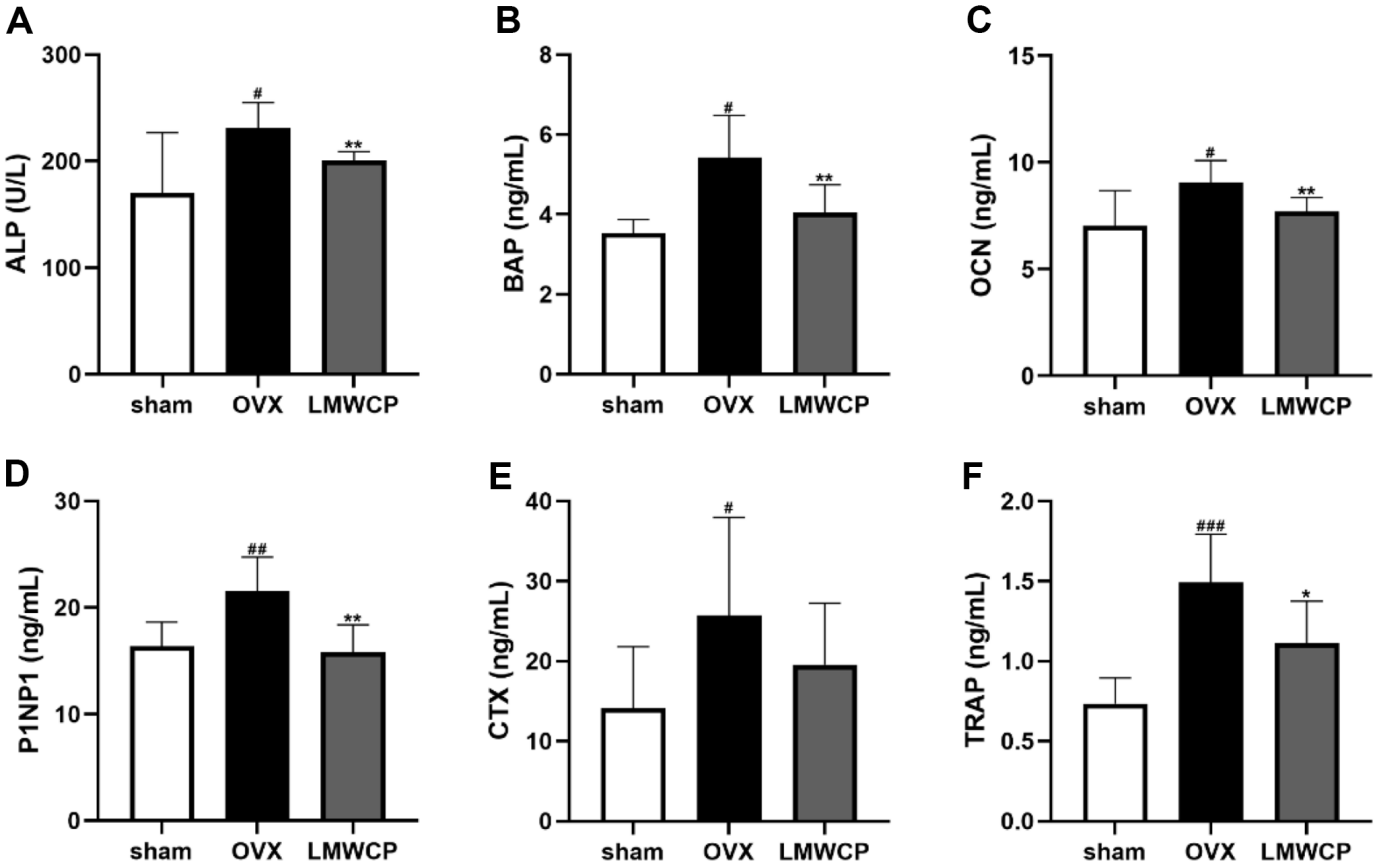
Effect of LMWCP on the serum levels of bone turnover markers. The ALP activities (**A**), BAP levels (**B**), OCN levels (**C**), P1NP levels (**D**), CTX levels (**E**), and TRAP levels (**F**) of sham, OVX, and OVX + LMWCP (at different LMWCP concentrations) rats were determined. Data represent the mean values of three independent experiments; #, *p* < 0.05; ##, *p* < 0.01; ###, *p* < 0.001 (when compared with the sham group); *, *p* < 0.05; **, *p* < 0.01 (when compared with the OVX group).

There are several markers in serum that indicate the bone turnover. Among them, ALP is one of the factors that represent the formation of osteoblasts, while BAP is the enzyme especially generated by osteoblasts. Moreover, OCN indicates the resorption activity of osteoblasts, while P1NP is an early parameter of the bone anabolic activity [29]. The administration of LMWCP has managed to attenuate the upregulation of ALP, BAP, OCN, and P1NP in the serum of OVX rats (Fig. 6A-6D). CTX and TRAP levels represent the resorption activity of osteoclasts. Both factors were found to be increased in the OVX group when compared to those of the sham group. When the LMWCP was administered to OVX rats, the serum levels of CTX and TRAP were found to be decreased (Fig. 6E and 6F).

## Discussion

Collagen hydrolysate has been shown to exert antiaging, antihypertensive, antiobesity, and wound healing effects [30]. Reports have also elucidated the role of collagen hydrolysate in skin aging [6] and cartilage formation [31], while its osteogenic effect has also been studied [32]. However, there is a lack of evidence regarding the oral administration of fish-deriving collagen hydrolysate on hematological factors and morphometric parameters in OVX rats. Therefore, in this study, we conducted in vitro and in vivo experiments to assess the effect of orally administered LMWCP on bone formation and morphogenesis.

The in vitro results obtained with the use of MC3T3-E1 cells suggested that LMWCP could increase ALP activity, stimulate mineralization, and induce collagen synthesis. The quantitative measurement of the ALP activity represents an early cell differentiation marker of osteoblastic cells, and its increased value is relevant to the ongoing differentiation of osteoblasts [33]. The formation of mineralized nodules represents an increased deposition of calcium. The phenotypic expression of an osteogenic tissue is its capability to form an ECM that can proceed into mineralization [34]. As collagen comprises 90% of the organic matrix of the bone, the synthesis of collagen is considered a critical parameter of osteoblast differentiation [35]. The effect of LMWCP on osteoblasts has been shown through the ALP activity, mineralization, and collagen deposition.

Being a renowned treatment for osteoporosis, estradiol (E2) is commonly used as a positive control in osteogenesis experiments. Studies that have used E2 as a positive control have produced similar results with those of this study [33, 36, 37], thereby supporting our data inferring that LMWCP can act similarly to E2. mRNA expression levels of bone metabolism markers such as OCN, OSX, BSP, and RUNX2 were increased in the presence of high concentration of LMWCP. We can therefore conclude that the LMWCP can stimulate the bone generation parameters and exert osteogenic effects on the studied cells. Moreover, the mRNA expression of *COL1A1* was found to be upregulated in MC3T3-E1 cells. One of the main constituents of LMWCP, Gly-Pro-Hyp, upregulated the expression of Col1A1 in MC3T3-E1 cells [1]. The upregulation of Col1A1 has also been speculated in human osteoblastic hFOB1.19 cells [10]. Moreover, in cells treated with 10 μg/ml of collagen tripeptide (which is contained at a level of over 15% w/w in LMWCP), type I collagen production was stimulated through the upregulation of *COL1A1* [10]. These findings indicating an increase in collagen synthesis were also confirmed by our in vivo data. We have assessed type I collagen density in OVX rats using immunohistochemistry. The result revealed a significant increase in type I collagen synthesis in both the femur and the lumbar vertebrae when OVX rats received LMWCP orally. Moreover, Tsuruoka *et al*. have suggested that the C-terminal collagen propeptide, which is procollagen containing an extended peptide that is synthesized to form collagen, was increased after the oral administration of collagen tripeptide in a fracture healing model of rats [10].

Our study also shows that LMWCP has a beneficial effect on the trabecular microarchitecture of the femur and the lumbar vertebrae. The BMD levels were found to be increased in both the femur and the lumbar vertebrae of the rats belonging to the OVX + LMWCP group, while those of the OVX group were found to be significantly decreased. BMD is a parameter that can predict the risk of bone fracture; therefore, the enhancement of its levels means that the LMWCP might be able to lower the possibility of developing osteoporosis. Other factors such as the BV/TV were also shown to be significantly increased in both bone tissues. The BV/TV values were similar in the femur and the lumbar vertebrae, but the SMI score was different. SMI is the index that shows the characteristic of trabeculae structure. As the value approached 0 and 3, trabeculae showed plate-like and rod-like structures. Based on these data, one can infer that the two bone tissues have different mechanical characteristics [38].

The hematological analysis revealed that the ALP, CTX, TRAP, BAP, OCN, and P1NP levels (all acting as bone turnover markers) were significantly higher in the OVX group when than in the sham group. Meanwhile, the levels of these same factors were found to decrease after a treatment with LMWCP. Several studies that have been conducted with the use of E2 have demonstrated a similar trend. During an assessment of Tantikanlayaporn *et al*., OCN, and TRAP activity in OVX rats, their levels were found to be decreased when the OVX rats were treated with E2 [39]. Since the bone turnover parameters are related to the suppression of the bone degradation rate, our results demonstrated that the LMWCP was able to decrease the bone turnover rate by repressing the osteoclast activity in the OVX rats. The ALP activity, a marker of the activity of osteoblasts, was significantly attenuated by the LMWCP treatment. Han *et al*. have also demonstrated the downregulation of the ALP activity after a treatment with E2 [40]. In addition, the bone specific ALP activity decreased in this study and E2-employing experiments [40]. Rissanen *et al*. have reported that P1NP was increased after an ovariectomy and decreased after the administration of E2 [41]. Our data also indicated a decrease in the levels of P1NP (which is a marker of bone formation) in the OVX rat model. To sum up, the levels of bone remodeling markers in the serum of LMWCP-treat OVX rats have exhibited the same behavior as those in the serum of E2-treated rats. Therefore, it can be assumed that LMWCP could be a possible candidate for the development of a diet-based preventive treatment for osteoporosis.

Previously, when a mouse received 900 mg/kg of a collagen hydrolysate comprising Gly-Pro-Hyp and was hydrolyzed with the same enzyme used herein, the maximum concentration (Cmax) of Gly-Pro-Hyp in plasma was 103.08 nmol/mL and the AUC was 2732.22 nmol∙min/g. Among the various peptides, Gly-Pro-Hyp was the one with the highest concentration. Furthermore, intact Gly-Pro-Hyp was degraded to Pro-Hyp, which is found in plasma and skin [1]. Yammamoto *et al*. [3] demonstrated that radiolabelled Gly-[^3^H]Pro-Hyp could be detected in the bone and bone marrow 24 h after oral administration. According to the kinetics of Gly-Pro-Hyp, this material would activate ECM synthesis, in line with the results of current study.

According to Naoki *et al* [10], LMWCP acts on Runx2 through osx, a known factor in osteoblast differentiation, regulating *Col1A1*, *BSP*, and *OCN*, major bone matrix protein genes. Our data are in line with the findings of Kim *et al*. [29], who used a porcine collagen hydrolysate in OVX rats. They have demonstrated that the collagen hydrolysate can stimulate the ERK pathway in order to induce collagen synthesis. LMWCP increased *col1a1* mRNA levels and collagen deposition in osteoblasts and type I collagen synthesis in OVX rats. This suggests that LMWCP plays a pivotal role in collagen synthesis by up-regulating the expression level of *col1a1* during bone formation. It is highly probable that the ERK pathway is involved in collagen synthesis by LMWCP. However, our data alone cannot fully explain the signaling pathway; therefore, further studies are required.

We have previously demonstrated that LMWCP has positive effects on the skin [15, 17] and the cartilage [18] through the activation of the synthesis of type I collagen and hyaluronic acid in skin fibroblasts and of type II collagen and aggrecan in chondrocytes, respectively. As LMWCP was able to stimulate major cells in the skin and the cartilage, it might be able to similarly stimulate the bone tissue cells. Functional peptides such as Gly-Pro-Hyp and Pro-Hyp can be absorbed after oral administration and are the main peptides that form collagen types I and II. Key pharmacokinetic parameters as Yazaki [42] has described, Gly-Pro-Hyp and Pro-Hyp are absorbed intact. Furthermore, Gly-Pro-Hyp administration increases Gly-Pro-Hyp and Pro-Hyp levels in plasma and skin. the Moreover, studies have demonstrated the action of Gly-Pro-Hyp and Pro-Hyp in osteoblasts and skin fibroblasts [1,43]. Therefore, we can hypothesize that Gly-Pro-Hyp and Pro-Hyp could act as inducers of the stimulation of collagen synthesis in tissues.

Overall, our study has shown that LMWCP can stimulate the differentiation of the bone, and can exert anabolic effects on bone formation. However, the elucidation of the exact mechanism of these effects requires further research.

## Conclusion

This study shows that the LMWCP exhibits a preventive effect against bone loss in MC3T3-E1 cells and OVX rats. We, herein, demonstrate that this material can suppress bone resorption and support bone formation. Therefore, LMWCP could be considered as a promising candidate for the development of a diet supplement that would support the prevention of osteoporosis.

## Supplemental Materials

Supplementary data for this paper are available on-line only at http://jmb.or.kr.



## Figures and Tables

**Table 1:** RT-qPCR primer sequences used in this study.

Gene	Forward (5'→3')	Reverse (5'→3')
BSP	AGG ACT GCC GAA AGG AAG GTT A	AGT AGC GTG GCC GGT ACT TAA A
COL1A1	GAG CGG AGT ACT GGA TCG	GCT TCT TTT CCT TGG GGT T
GAPDH	ATC CCA TCA CCA TCT TCC AGG AG	CCT GCT TCA CCA CCT TCT TGA TG
OCN	GAG GAC CAT CTT TCT GCT CAC TCT	TTA TTG CCC TCC TGC TTG GA
OSX	AGG AGG CAC AAA GAA GCC ATA C	AGG GAA GGG TGG GTA GTC ATT
RUNX2	GCA CAA ACA TGG CCA GAT TCA	AAG CCA TGG TGC CCG TTA G

Gene abbreviations: BSP, bone sialoprotein; COL1A1, collagen type 1, alpha 1; GAPDH, glyceraldehyde-3-phosphate dehydrogenase; OCN, osteocalcin; OSX, osterix; RUNX2, Runt-related transcription factor 2.
