# Supplementary material for: In Vitro and In Vivo Bone-Forming Effect of a Low-Molecular-Weight Collagen Peptide
Source: J Microbiol Biotechnol. 2023 Nov 20;34(2):415–24. doi: 10.4014/jmb.2307.07017 (PMC10940753; doi:10.4014/jmb.2307.07017)
Supplement: Supplementary file 1 [file jmb-34-2-415-supple.pdf]

J. Microbiol. Biotechnol.
https://doi.org/10.4014/jmb.2307.07017

Supplementary Figure 1.

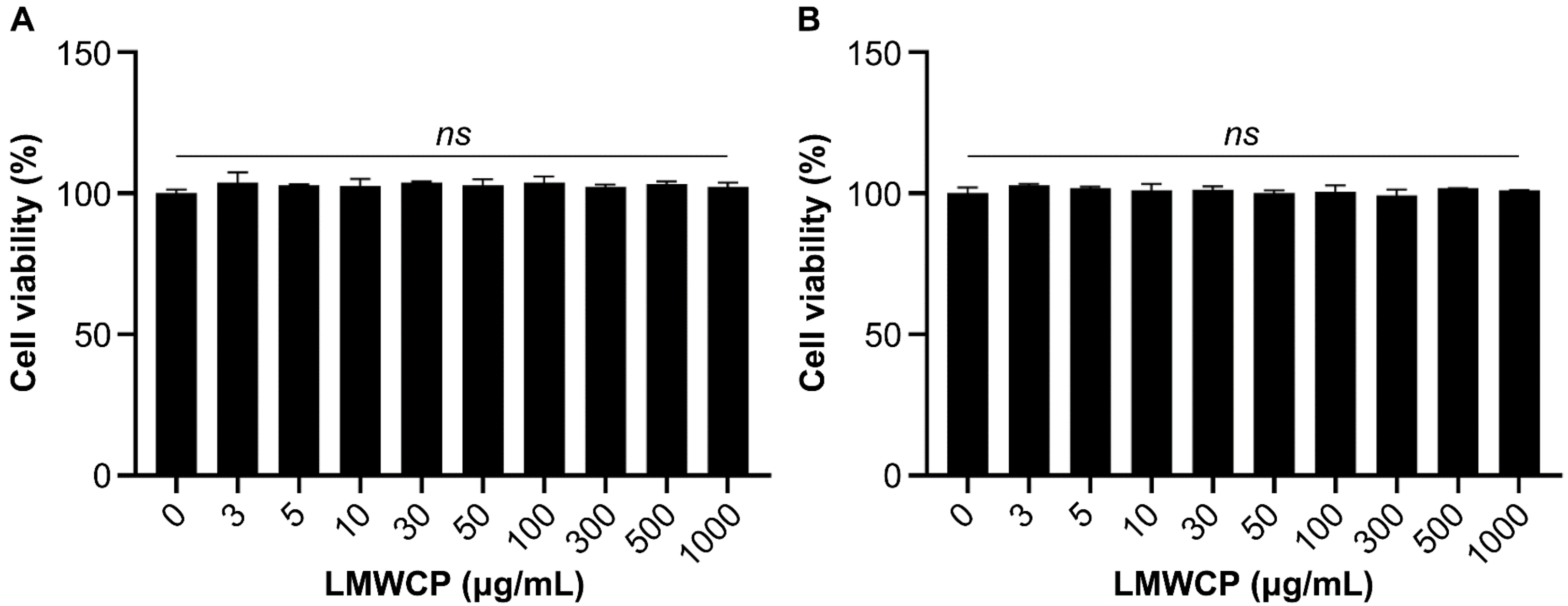

Effect of LMWCP on the cell viability of MC3T3-E1 cells. MC3T3-E1 cells were treated with various concentrations of LMWCP (3, 5, 10, 30, 50, 100, 300, 500, and 1,000 μg/mL) for 24 h (A) and 48 h (B). The changes of cell viability as a result of the LMWCP treatment were detected using an EZ-Cytox kit assay. Data represent the mean values of three independent experiments; ns, no significant difference (when compared with the control group).

Supplementary Figure 2.

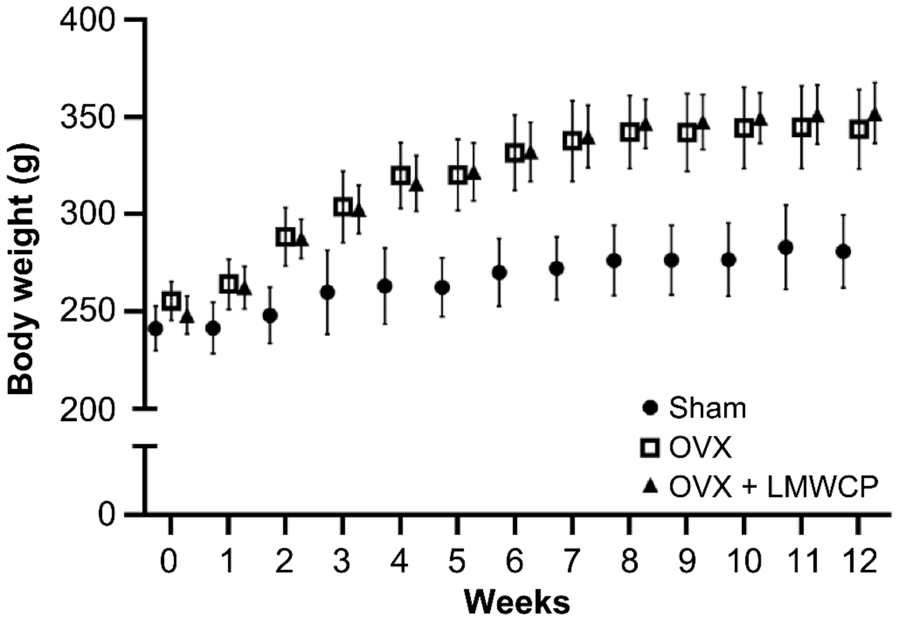

Effect of LMWCP on the body weight of OVX rats. The body weights of the rats were measured weekly on a specific time of the day. No significant differences were observed.
